# Supplementary material for: The feasibility of a visuo-cognitive training intervention using a mobile application and exercise with stroboscopic glasses in Parkinson’s: Findings from a pilot randomised controlled trial
Source: PLOS Digit Health. 2024 Dec 18;3(12):e0000696. doi: 10.1371/journal.pdig.0000696 (PMC11654989; doi:10.1371/journal.pdig.0000696)
Supplement: S1 File — (DOCX) [file pdig.0000696.s001.docx]

**Supporting Information File 1: CONSORT checklist of information to include when reporting a pilot trial***

| **Section/topic and item No** | **Standard checklist item** | **Extension for pilot trials** | **Page no. where item is reported** |
| --- | --- | --- | --- |
| **Title and abstract** | | | |
| 1a | Identification as a randomised trial in the title | Identification as a pilot or feasibility randomised trial in the title | 1 |
| 1b | Structured summary of trial design, methods, results, and conclusions (for specific guidance see CONSORT for abstracts) | Structured summary of pilot trial design, methods, results, and conclusions (for specific guidance see CONSORT abstract extension for pilot trials) | 1 |
| **Introduction** | | | |
| Background and objectives: | | | |
| 2a | Scientific background and explanation of rationale | Scientific background and explanation of rationale for future definitive trial, and reasons for randomised pilot trial | 5 |
| 2b | Specific objectives or hypotheses | Specific objectives or research questions for pilot trial | 5 |
| **Methods** | | | |
| Trial design: | | | |
| 3a | Description of trial design (such as parallel, factorial) including allocation ratio | Description of pilot trial design (such as parallel, factorial) including allocation ratio | 6  S1 |
| 3b | Important changes to methods after trial commencement (such as eligibility criteria), with reasons | Important changes to methods after pilot trial commencement (such as eligibility criteria), with reasons | N/A |
| Participants: | | | |
| 4a | Eligibility criteria for participants |  | S1 |
| 4b | Settings and locations where the data were collected |  | 6 |
| 4c |  | How participants were identified and consented | 6 |
| Interventions: | | | |
| 5 | The interventions for each group with sufficient details to allow replication, including how and when they were actually administered |  | 6-7  S3 |
| Outcomes: | | | |
| 6a | Completely defined prespecified primary and secondary outcome measures, including how and when they were assessed | Completely defined prespecified assessments or measurements to address each pilot trial objective specified in 2b, including how and when they were assessed | 7-10  S1 |
| 6b | Any changes to trial outcomes after the trial commenced, with reasons | Any changes to pilot trial assessments or measurements after the pilot trial commenced, with reasons | N/A |
| 6c | If applicable, prespecified criteria used to judge whether, or how, to proceed with future definitive trial |  | N/A |
| Sample size: | |  |  |
| 7a | How sample size was determined | Rationale for numbers in the pilot trial | S1 |
| 7b | When applicable, explanation of any interim analyses and stopping guidelines |  | N/A |
| Randomisation: |  |  |  |
| Sequence generation: | |  |  |
| 8a | Method used to generate the random allocation sequence |  | S1 |
| 8b | Type of randomisation; details of any restriction (such as blocking and block size) | Type of randomisation(s); details of any restriction (such as blocking and block size) | S1 |
| Allocation concealment mechanism: | |  |  |
| 9 | Mechanism used to implement the random allocation sequence (such as sequentially numbered containers), describing any steps taken to conceal the sequence until interventions were assigned |  | S1 |
| Implementation: |  |  |  |
| 10 | Who generated the random allocation sequence, enrolled participants, and assigned participants to interventions |  | S1 |
| Blinding: |  |  |  |
| 11a | If done, who was blinded after assignment to interventions (eg, participants, care providers, those assessing outcomes) and how |  | S1  23 |
| 11b | If relevant, description of the similarity of interventions |  | 7 |
| Analytical methods: |  |  |  |
| 12a | Statistical methods used to compare groups for primary and secondary outcomes | Methods used to address each pilot trial objective whether qualitative or quantitative | 7-11 |
| 12b | Methods for additional analyses, such as subgroup analyses and adjusted analyses | Not applicable | N/A |
| **Results** Participant flow (a diagram is strongly recommended): | | | |
| 13a | For each group, the numbers of participants who were randomly assigned, received intended treatment, and were analysed for the primary outcome | For each group, the numbers of participants who were approached and/or assessed for eligibility, randomly assigned, received intended treatment, and were assessed for each objective | 11  Fig. 2 |
| 13b | For each group, losses and exclusions after randomisation, together with reasons |  | 11  Fig. 2 |
| Recruitment: | | | |
| 14a | Dates defining the periods of recruitment and follow-up |  | 11 |
| 14b | Why the trial ended or was stopped | Why the pilot trial ended or was stopped | 11 |
| Baseline data: | | | |
| 15 | A table showing baseline demographic and clinical characteristics for each group |  | 11  Table 1 |
| Numbers analysed: | | | |
| 16 | For each group, number of participants (denominator) included in each analysis and whether the analysis was by original assigned groups | For each objective, number of participants (denominator) included in each analysis. If relevant, these numbers should be by randomised group | 11-15 |
| Outcomes and estimation: | | | |
| 17a | For each primary and secondary outcome, results for each group, and the estimated effect size and its precision (such as 95% confidence interval) | For each objective, results including expressions of uncertainty (such as 95% confidence interval) for any estimates. If relevant, these results should be by randomised group | 11-15  Tables 4 |
| 17b | For binary outcomes, presentation of both absolute and relative effect sizes is recommended | Not applicable | N/A |
| Ancillary analyses: | | | |
| 18 | Results of any other analyses performed, including subgroup analyses and adjusted analyses, distinguishing prespecified from exploratory | Results of any other analyses performed that could be used to inform the future definitive trial | 20 |
| Harms: | | | |
| 19 | All important harms or unintended effects in each group (for specific guidance see CONSORT for harms) |  | 12-14 |
| 19a |  | If relevant, other important unintended consequences | N/A |
| **Discussion** | | | |
| Limitations: | | | |
| 20 | Trial limitations, addressing sources of potential bias, imprecision, and, if relevant, multiplicity of analyses | Pilot trial limitations, addressing sources of potential bias and remaining uncertainty about feasibility | 21 |
| Generalisability: | | | |
| 21 | Generalisability (external validity, applicability) of the trial findings | Generalisability (applicability) of pilot trial methods and findings to future definitive trial and other studies | 15-21 |
| Interpretation: | | | |
| 22 | Interpretation consistent with results, balancing benefits and harms, and considering other relevant evidence | Interpretation consistent with pilot trial objectives and findings, balancing potential benefits and harms, and considering other relevant evidence | 15-21 |
| 22a |  | Implications for progression from pilot to future definitive trial, including any proposed amendments | 16, 17, 20, 21 |
| **Other information** | | | |
| Registration: | | | |
| 23 | Registration number and name of trial registry | Registration number for pilot trial and name of trial registry | 3, 6 |
| Protocol: | | | |
| 24 | Where the full trial protocol can be accessed, if available | Where the pilot trial protocol can be accessed, if available | 6  S1 |
| Funding: | | | |
| 25 | Sources of funding and other support (such as supply of drugs), role of funders |  | 23-24 |
| 26 | Ethical approval or approval by research review committee, confirmed with reference number |  | 5-6 |

**CONSORT for abstracts extension for pilot randomised trials***

| **Abstract -** | **Item** | **Extension for pilot trials** | **Reported** |
| --- | --- | --- | --- |
| **Title.** The feasibility of a visuo-cognitive training intervention using a mobile application and exercise with stroboscopic glasses in Parkinson’s: Findings from a pilot randomised controlled trial  **Background**. There is currently no pharmacological treatment for visuo-cognitive impairments in Parkinson’s disease. Alternative strategies are needed to address these non-motor symptoms given their impact on quality of life. Novel technologies have potential to deliver multimodal rehabilitation of visuo-cognitive dysfunction, but more research is required to determine their feasibility in Parkinson’s.  **Objective**. To determine the feasibility and preliminary efficacy of a home-based, technological visuo-cognitive training (TVT) intervention using a mobile application and exercise with stroboscopic glasses compared to non-technological care in people with Parkinson’s.  **Methods**. This 18-month, parallel, two-arm pilot trial took place between July 2021-December 2022. Participants were community-dwelling individuals with a diagnosis of Parkinson’s, aged over 50 years. Participants were randomly allocated to one of two active four-week interventions, TVT (n=20) or standard care (SC) (n=20). A physiotherapist delivered 8 home visits over 4 weeks, lasting 45-60 mins. Participants were evaluated at baseline and then on completion of the intervention. Primary outcomes were feasibility of the study design and intervention (recruitment/retention, adherence, assessment time scale, equipment and safety). Exploratory outcomes included assessments of cognitive, visual, clinical and motor function. (Blinding of participants was not possible due to the nature of the intervention).  **Results**. The recruitment rate was 60% (40/67), and the retention rate was 98% (39/40). Adherence to both arms of the intervention was high, with participants attending 98% of visits in the TVT group and 96% of visits in the SC group. 35% (9/20) of participants in the TVT group experienced mild symptoms associated with use of the stroboscopic glasses which included dizziness, queasiness and unsteadiness. There were minimal between group differences, with both interventions having positive effects on a variety of clinical, cognitive, and physical performance outcomes.  **Conclusions**. Our findings suggest that home-based TVT with a physiotherapist is feasible in people with Parkinson’s and could provide an alternative approach to addressing cognitive and motor dysfunction in this population. We make recommendations for future trials and invite ensuing studies to improve upon the design and utilise stroboscopic visual training and digital tools to investigate this emerging area of multimodal rehabilitation.  **Trial Registration**. ISRCTN46164906.  **Trial Funding**. Northumbria University PhD studentship in collaboration with Senaptec Ltd. (Beaverton, Oregon, USA). | Title | *Identification of study as a randomised pilot trial* | ✓ |
|  | Trial design | *Description if pilot trial design* | ✓ |
|  | METHODS |  |  |
|  | Participants | *Eligibility criteria for participants and the settings where the pilot trial was conducted* | ✓ |
|  | Interventions | *Interventions intended for each group* | ✓ |
|  | Objective | *Specific objectives of the pilot trial* | ✓ |
|  | Outcome | *Pre-specified assessment or measurement to address the pilot trail objective(s)* | ✓ |
|  | Randomisation | *How participants were allocated to interventions* | ✓ |
|  | Blinding (masking) | *Whether or not participants, care givers, and those assessing the objectives were blinded to group assignment* | ✓ |
|  | RESULTS |  |  |
|  | Numbers randomised | *Number of participants screened and randomised to each group for the pilot trial objective(s)* | ✓ |
|  | Recruitment | *Trial status (for conference abstracts)* | N/A |
|  | Numbers analysed | *Number of participants analysed in each group for the pilot trial objective(s)* | ✓ |
|  | Outcome | *Results for the pilot trial objective(s); including any expressions of uncertainty* | ✓ |
|  | Harms | *Important adverse events or side effects* | ✓ |
|  | Conclusions | *General interpretation of the results of the pilot trial and their implications for the future definitive trial* | ✓ |
|  | Trial registration | *Registration number for pilot trial and name of trail register* | ✓ |
|  | Funding | *Source of funding for pilot trial* | ✓ |
|  |  | | |

*Adapted from: CONSORT 2010 statement: extension to randomised pilot and feasibility trials, Eldridge et al. 2016
