# Supplementary material for: The feasibility of a visuo-cognitive training intervention using a mobile application and exercise with stroboscopic glasses in Parkinson’s: Findings from a pilot randomised controlled trial
Source: PLOS Digit Health. 2024 Dec 18;3(12):e0000696. doi: 10.1371/journal.pdig.0000696 (PMC11654989; doi:10.1371/journal.pdig.0000696)
Supplement: S3 File — (DOCX) [file pdig.0000696.s003.docx]

**Follow-up assessment in Gait Laboratory (week 5)**

**Baseline assessment in Gait Laboratory (week 0)**

**Randomisation**

**(n=40)**

**Home-based TVT Intervention**

**(weeks 1-4)**

Participants received twice weekly visits lasting approx. one hour from a physiotherapist over four weeks.

Each session included a series of visuo-cognitive training drills performed on a mobile tablet device and exercises whilst wearing stroboscopic glasses.

Activities were supervised at all times by a qualified physiotherapist from the research study team.

On the final visit, a semi-structured interview was conducted to explore participants’ experiences of the technological intervention.

**Home-based Standard care Intervention (weeks 1-4)**

Participants received twice weekly visits lasting approx. one hour from a physiotherapist over four weeks.

Each session included a series of paper-based visuo-cognitive training tasks and seated game activities plus exercises under normal visual conditions.

Activities were supervised at all times by a qualified physiotherapist from the research study team.

**Supporting Information File 3**. Outline of interventions
