# Supplementary material for: The feasibility of a visuo-cognitive training intervention using a mobile application and exercise with stroboscopic glasses in Parkinson’s: Findings from a pilot randomised controlled trial
Source: PLOS Digit Health. 2024 Dec 18;3(12):e0000696. doi: 10.1371/journal.pdig.0000696 (PMC11654989; doi:10.1371/journal.pdig.0000696)
Supplement: S4 File — (DOCX) [file pdig.0000696.s004.docx]

**Supporting Information File 4.** Description of the visuo-cognitive training interventions using TIDieR framework

|  | |
| --- | --- |
| **TIDieR**  **component** | **Description** |
| Why (rationale) | Visual, cognitive and motor systems all decline with PD pathology and are difficult to treat. Combining multiple digital technologies (i.e. stroboscopic glasses, mobile applications) into a multi-modal technological visuo-cognitive training (TVT) programme may provide effective rehabilitation for people with PD. It is not yet known whether these approaches are effective or how they compare to standard (traditional) approaches to rehabilitation. |
| What (materials) | **TVT Intervention**   - Stroboscopic glasses: Senaptec Strobe Training Eyewear with lenses that flicker between clear and opaque (https://senaptec.com/products/senaptec-strobe) - Sensory training application (https://senaptec.com/products/senaptec-app) delivered via tablet: Apple iPadOS 16.1.1 (20B101) 8^th^ Gen 128GB   **Standard Intervention**   - Pen/paper-based activities adapted from commonly used visuo-cognitive training activities used in clinical practice. - Commercially available games were used to offer comparable challenges to the app-based training drills.   Exercise equipment used by participants in both groups during throwing/catching/reaction time tasks): Lightweight ball (20cm diameter); bean bags; small plastic airflow balls |
| What (procedures) | A selection of visuo-cognitive training activities delivered via mobile application (TVT group) or pen/paper/game-based activities (approx. 15-20 mins) (SC group) plus throwing, catching, balance exercises delivered with (TVT) or without (SC) intermittent visual occlusion (up to 20 mins per session). |
| Who provided | A research physiotherapist with specialist neurorehabilitation skills and over 15 years clinical experience alongside research experience with people with PD and older adults. |
| How (delivery) | Two TVT sessions per week for 4 weeks or two SC sessions per week for 4 weeks. All sessions were delivered and supervised by the provider above. |
| Where | Participants’ homes (and one in University Gait Laboratory due to personal circumstance) |
| When and how much | Eight x 45-60 minute sessions (two per week for 4 weeks for each arm of intervention) |
| Tailoring | **TVT Intervention**   - Throwing/catching/balance activities were delivered with intermittent visual occlusion via strobe glasses (in sitting/standing depending on participant ability and exercise tolerance). Challenge was progressed by increasing length of opaque phase on stroboscopic glasses so that participants had to work with less visual information as well as increasing speed/intensity, duration or amount of each exercise. - App-based drills were programmed to automatically increase in difficulty as visuo-motor skills improve.   **Standard Intervention**   - The same throwing/catching/balance activities were delivered with uninterrupted vision (in sitting/standing depending on participant ability and exercise tolerance). Challenge was progressed by increasing speed/intensity, duration or amount of each exercise. - Pen/paper and game-based activities were progressed by increasing the challenge/complexity level of tasks such as Tangrams/shape matching, adding increased rate of metronome beats to visual naming tasks, and counting number of errors (e.g on buzz wire) or adding time constraints/time goals to completion. |
| Modifications | The study began as a cross-over design and was changed to a parallel-group design for pragmatic reasons. |
| How well (planned) | The provider was present for every home-based intervention and documented session content for each visit, including nature of exercises, level of strobe setting and length of training tolerated. The provider also made written notes about participant response to exercises/activities, level of engagement and any feedback given by participants in relation to the training. |

Adapted from Das et al. 2023 doi: <https://doi.org/10.1371/journal.pone.0285100.g001>
