# Supplementary material for: The feasibility of a visuo-cognitive training intervention using a mobile application and exercise with stroboscopic glasses in Parkinson’s: Findings from a pilot randomised controlled trial
Source: PLOS Digit Health. 2024 Dec 18;3(12):e0000696. doi: 10.1371/journal.pdig.0000696 (PMC11654989; doi:10.1371/journal.pdig.0000696)
Supplement: Attachment — Submitted filename: Response to reviewers.docx [file pdig.0000696.s005.docx]

PDIG-D-24-00019
The feasibility of a visuo-cognitive training intervention using a mobile application and exercise with stroboscopic glasses in Parkinson’s: Findings from a pilot randomised controlled trial, PLOS Digital Health

We would like to thank the editors and the reviewers for their hard work in reviewing our manuscript. We have added our reviewer responses to the table below, which are shown as tracked changes highlighted in yellow within the manuscript.

| **Editorial queries** | **Author response** |
| --- | --- |
| A major question is whether your  currently presented work is substantially different than the already previously published work (in this and other journals). | Our feasibility and preliminary efficacy findings have not been published in any other journals; preliminary findings were presented in abstract format by poster presentation at the Movement Disorders Congress in Copenhagen in 2023.  We have published some initial qualitative findings relating to a subset of participants who took part in an earlier phase of the study ([The integration of technology into a home-based visuo-cognitive training intervention for people with Parkinson’s: Is the future digital? \| PLOS ONE](https://journals.plos.org/plosone/article?id=10.1371/journal.pone.0285100)), and these are referenced in our main results paper to add context to the feasibility findings presented here. |
| Please clarify in your manuscript what the new contributions are and provide more details in methods, results and discussion to support those claims.  Please also add a rationale, supported by existing literature, that would have lead to your feasibility study. | Thank you for identifying these gaps. We have added the following statement (highlighted) in the conclusion to emphasise what the new contributions to research are:  “The current study responds to the need for new and improved interventions to address visuo-cognitive dysfunction and contributes to the evidence base for multimodal approaches to rehabilitation in PD. Furthermore, this study not only provides a novel insight into the use of strobe glasses as an adjunct to exercise in PD, it is also the first time that a comprehensive technological visuo-cognitive training programme has been used in this population. Our study demonstrates that home-based TVT is feasible with therapy support and the components of the intervention warrant further exploration.”  We have included the following text, supported by literature, to justify the rationale for our study:  (Line 119): “Visuo-cognitive impairments have been associated with motor deficits in people with PD, specifically postural instability and gait impairment(5, 17-20). This is further complicated by the fact that deficits in the ability to use proprioceptive feedback in people with PD result in greater reliance on impaired visuo-cognitive function to carry out motor tasks (5, 21-25). The combination of visuo-cognitive deficits and increased dependence on faulty visuo-cognitive information has a significant impact on motor symptoms in people with PD, particularly gait and balance(24, 26, 27). Specifically, visuo-cognitive impairments reduce the ability to compensate for underlying motor dysfunction in Parkinson’s, which can lead to a decline in daily activities, increased risk of falls and reduced quality of life(28-30).  There is a lack of pharmacological treatment options for visuo-cognitive deficits(31). Previous studies have shown some improvement in visuo-cognitive function in people with PD using clinical interventions, such as eye movement training and exercises that use paper-based or gamified activities (i.e., throwing and catching, hand eye co-ordination tasks etc.)(32-34). However, the treatments described in these trials often involve participants having to attend multiple training sessions per week at a laboratory/clinic/hospital or performing repetitive activities, such as pencil push ups several times per day(34). These studies have also been limited by small sample sizes, strict inclusion criteria, and the burdensome protocols that resulted in significant drop-out rates and low participant uptake, which all limit the generalisability to clinical practice.  As a result of these limitations, there is growing interest in the development of visuo-cognitive training programmes provided via digital technologies to offer an alternative approach to traditional rehabilitation interventions. These can be carried out using modern digital technologies, such as computer or mobile applications(35-42). Technologies can offer potential advantages over paper-based tasks in that they can provide real-time feedback, allow modulation of task progression and automatic recording of session parameters(43). In addition to computerised training applications, novel devices also have the potential to enhance traditional visuo-cognitive training approaches. For example, Shalmoni & Kalron (2020) demonstrated that stroboscopic eyewear could enhance information processing speed immediately after training in people with multiple sclerosis(44).  As awareness of how visuo-cognitive processes directly and indirectly influence motor task performance in PD increases, interest in the potential of multimodal visuo-cognitive training strategies to simultaneously target visual, cognitive and motor abilities has grown(8-10, 45). To date, no studies have examined the combined use of stroboscopic visual training with a cognitive training application in PD (here termed technological visuo-cognitive training (“TVT”)). Therefore, the aims of the present study were to; 1) Investigate the feasibility of home-based TVT compared to non-technological visuo-cognitive training (here termed standard care (“SC”))SC in PD; 2) Provide initial efficacy data of home-based TVT compared to SC in PD; and 3) To provide data to inform future larger scale clinical trials.” |
| On a last note, there is the open questions why the authors did not try to submit these study results in the same journal where they published the protocol? | Unfortunately, it is not always possible to publish clinical trial results in the same journal as the published protocol, as not all journals accept protocol submissions. Our choice of journal is appropriate in line with PLOS recommendations, as we used the “PLOS journal selector” function accessed via the PLOS website (<https://plos.org/your-journal-options/>) to help us make our initial decision to publish our protocol paper in PLOS One as other PLOS journals do not accept protocols. PLOS Digital Health is a more appropriate journal to publish our study findings because we were reporting on novel digital technologies in healthcare. |

| Reviewer #1 Feedback summary:  The current manuscript seems to be the second installment of a previously published research proposal. Therefore reviewing this manuscript required reading through the previous published paper in detail as well, and it would seem that readers will need to connect the two in order to have a full understanding of the project. While overall the research question and goals are interesting and the methodology is presented well with satisfactory detail, there is a lack of connection on the importance or rationale for using the technology based program. Some specific questions are below. | |
| --- | --- |
| Reviewer questions | Response |
| 1. What is the benefit of the mobile device/technology over and above the standard pen and paper or 'real world' tasks? | There are limited papers comparing technology-based versus paper-based cognitive training in clinical populations, and those that have been done have shown mixed results. Some suggest that technology-based cognitive training offers advantages over standard pen and paper tasks, by providing real-time feedback, automatic task progression and recording and storage of training outcomes (Bernini, Panzarasa et al. 2021). Other studies have revealed both approaches can have beneficial effects (Georgopoulou, Nousia et al. 2023) but it is clear that more research is needed in this area.  We therefore do not currently know whether there is an advantage of technology over and above standard care/real world tasks in people with Parkinson’s, which is one of the primary reasons we chose to have an active “standard care” parallel group comparator rather than an inactive control arm.  We thank you for your comment and appreciate that this was not made clear in the manuscript. We have included the following text in the introduction to reflect this:  (Line 144): “Technologies can offer potential advantages over paper-based tasks in that it can provide real-time feedback, allow modulation of task progression and automatic recording of session parameters(43). In addition to computerised training applications, novel devices also have the potential to enhance traditional visuo-cognitive training approaches.” |
| 2. Why is the use of stroboscopic glasses considered a particular strength in the study? This was inadequately addressed in the previous publication as well. What would this kind of training add over and above what may already be done in standard care "SC"? Where would you expect the impact to be (e.g. what variables or measures would this have particular influence on)? Did you see any evidence of this? Other than stating it is a strength, in reading the manuscript I do not see where that strength may be or where the results support this statement. | Following publication of our original protocol in 2022, the authors published a review of the literature relating to the use of stroboscopic glasses in neurological populations (<https://pubmed.ncbi.nlm.nih.gov/37611053/>)  The review highlights the potential benefits of using stroboscopic visual training (SVT) in clinical practice but also emphasises where there are gaps in the current research. Our review revealed that although there is still no consensus with regard to the optimal length of training with strobes and the most effective strobe frequency at which to train, SVT can enhance visual skills with some evidence for training effects translating to balance and physical performance in both healthy and clinical populations. We have added reference to this review in the manuscript, and thank the reviewer for highlighting the omission.  We feel that the true strength of our study lies in the combination of strobes with the mobile application (TVT package) which has the potential to provide more comprehensive visuo-cognitive training than is possible with standard care. We have therefore reframed our strengths section to reflect this:  (Line 561): “The greatest strength of this study is the combined use of stroboscopic glasses with a mobile application to provide comprehensive visuo-cognitive rehabilitation. This is more representative of what would be carried out in multidisciplinary clinical practice(91).”  We value the reviewer’s comments in relation to the use of the strobe glasses being considered a strength and amended the wording to reflect this:  (Line 585): “This study is novel in lies in its use of stroboscopic technology as part of a multimodal rehabilitation intervention for people with PD. Stroboscopic training has never previously been used in this population, despite evidence for its potential benefits in clinical practice(47).” |
| 3. I realize this is a feasibility study and it seems that the result of the study is yes, technology can be added in a feasible way to at home training. But again why is this desired? What benefit does this have over and above SC? Based on some of the QOL data of this study it seems that potentially the SC has more benefits if we consider QOL and potential social engagement of the PwP in their training. This result is downplayed a little with more emphasis on the benefits of the technology. | Thank you for raising this. As you noted, this is primarily a feasibility study and we have therefore tried not to overestimate our efficacy findings. We also acknowledge that there will always be an inherent difficulty with interpretation of the data given the multiple testing that took place.  We did feel it was important to include efficacy data given the wealth of objective measurements that were undertaken and to contribute to the reader’s understanding of how we determined future sample size estimations and which outcomes should be used to capture change in future studies. We acknowledged that the data should be “be treated with caution due to the potential problems arising from the small sample sizes and low statistical power” (line 624) and only made intext reference to effect sizes rather than p values.  We are grateful for your comments, as it was not our intention to overstate our findings in relation to the technology. We have amended some of the text in the discussion section to further emphasise the feasibility aspects of this study (see tracked changes in manuscript).  We have also added the following text (see highlighted, line 506) into the discussion section around the QoL outcomes to emphasise the need to acknowledge the benefits of social engagement in rehabilitative practices:  “Health-related QoL as measured with the PDQ39 showed positive trends in the SC group compared to the TVT group. The nature of the training may have contributed to this finding, as participants were actively engaging with the researcher during the paper-based activities and games, whereas those in the TVT group were working on an iPad with less interaction(Georgopoulou, Nousia et al. 2023). This is contextualised by data from the interviews which showed the value that people with PD placed on the therapy interaction that accompanied the training sessions (qualitative findings published elsewhere)(Das, Barry et al. 2023). Future work needs to determine how much influence social engagement has on the uptake and integration of technology into rehabilitative practices(Das, Barry et al. 2023).  (Line 557): “Inclusion of QoL measures and fear of falling should also be considered in future studies, given the difference in findings between the two intervention approaches.” |
| 4. In addition to the above, the stroboscopic goggles may also have contributed to some motion sickness related discomfort. Again, this brings me to question why use these if there is no further benefit to SC training but brings with it added risk? | Thank you for highlighting this. Previous literature does suggest that stroboscopic visual training can enhance visual skills with some evidence for training effects translating to balance and physical performance in both healthy and clinical populations (Shalmoni and Kalron 2020, Symeonidou and Ferris 2022), but we cannot comment on the efficacy (or lack of) of the strobe training in isolation due to the multimodal nature of our intervention. We acknowledge this as a limitation and have included the following text:  (Line 611): “Given the potential to experience negative symptoms as a result of the stroboscopic effect, it is essential that future trials are conducted to establish the clinical efficacy of stroboscopic training in PD (and whether it is superior to exercise under normal visual conditions as part of a TVT package) in order to facilitate future user-adoption. Negative symptoms may prove to be an exclusion criteria for use of the strobes in clinical practice, or may indicate the need to use lower strobe settings to build up tolerance. Future work should explore the impact of stroboscopic training length on symptom severity over time, and establish guidelines for their use in clinical practice.” |

Reviewer #2: This paper presents preliminary work toward a larger goal of understanding risks and benefits of a novel visual-cognitive training intervention to improve balance and function in individuals with Parkinson's disease (PD). It's strength is that the authors do not attempt to overestimate their findings, showing the feasibility of the intervention but understanding their preliminary results to not show improvement over standard care. The manuscript is well-written, with appropriate detail in both methodology and results. There are no issues noted in the writing and figures are clear and add to the paper. The only minor issue is that there could be more discussion about cost-benefit analysis and the role of the therapist for each of the treatments. It appears that the TVT intervention may require greater therapist involvement (given some increase in side-effects and need for treatment modifications in this group) which would raise the cost of providing this treatment compared to usual care. This is particularly relevant if the goal is an in-home training program. Other than that, the paper adds new information to the field of visual-motor training in PD and addresses an important, unmet need in this population.

| Reviewer comment | Response |
| --- | --- |
| The only minor issue is that there could be more discussion about cost-benefit analysis and the role of the therapist for each of the treatments. It appears that the TVT intervention may require greater therapist involvement (given some increase in side-effects and need for treatment modifications in this group) which would raise the cost of providing this treatment compared to usual care. This is particularly relevant if the goal is an in-home training program | The authors thank Reviewer 2 for their feedback and for raising this very pertinent issue. Unfortunately, it was beyond the scope of this study to consider a cost evaluation of the interventions, and we agree that this is a limitation of the current study. We have made reference to the cost implications for conducting future studies:  (Line 549): “Future studies would need to consider the cost implications of factoring in multisite data collection to ensure availability of staff and adequate equipment to deliver the face-to-face intervention.”  and also included the following text in the discussion section to acknowledge the implications of needing a therapist present to deliver the TVT intervention:  (Line 577): “Due to the novel, exploratory nature of this study, a therapist was in attendance at every session. Moving forwards, it will be necessary to consider how much therapist involvement is needed to deliver an exercise programme using novel technology rather than SC. This is particularly relevant if the ultimate goal is to facilitate self-management(95). Future studies using TVT should therefore show the costs and outcomes adjusted for differential resource needs in the context of rehabilitation for people with PD(96, 97).” |

Bernini, S., S. Panzarasa, M. Barbieri, E. Sinforiani, S. Quaglini, C. Tassorelli and S. Bottiroli (2021). "A double-blind randomized controlled trial of the efficacy of cognitive training delivered using two different methods in mild cognitive impairment in Parkinson’s disease: preliminary report of benefits associated with the use of a computerized tool." Aging Clinical and Experimental Research **33**(6): 1567-1575.

Das, J., G. Barry, R. Walker, R. Vitorio, R. Morris and S. Stuart (2023). "The integration of technology into a home-based visuo-cognitive training intervention for people with Parkinson's: Is the future digital?" PLoS One **18**(6): e0285100.

Georgopoulou, E. N., A. Nousia, V. Siokas, M. Martzoukou, E. Zoupa, L. Messinis, E. Dardiotis and G. Nasios (2023). "Computer-Based Cognitive Training vs. Paper-and-Pencil Training for Language and Cognitive Deficits in Greek Patients with Mild Alzheimer's Disease: A Preliminary Study." Healthcare (Basel) **11**(3).

Shalmoni, N. and A. Kalron (2020). "The immediate effect of stroboscopic visual training on information-processing time in people with multiple sclerosis: an exploratory study." Journal of Neural Transmission: 1-7.

Symeonidou, E.-R. and D. P. Ferris (2022). "Intermittent Visual Occlusions Increase Balance Training Effectiveness." Frontiers in human neuroscience **16**: 748930-748930.
